# A Strategy to Reduce Bias of Entropy Estimates in Resting-State fMRI Signals

**DOI:** 10.3389/fnins.2018.00398

**Published:** 2018-06-13

**Authors:** Albert C. Yang, Shih-Jen Tsai, Ching-Po Lin, Chung-Kang Peng

**Affiliations:** ^1^Division of Interdisciplinary Medicine and Biotechnology, Beth Israel Deaconess Medical Center, Harvard Medical School, Harvard University, Boston, MA, United States; ^2^Institute of Brain Science, National Yang-Ming University, Taipei, Taiwan; ^3^Department of Psychiatry, Taipei Veterans General Hospital, Taipei, Taiwan; ^4^Division of Psychiatry, School of Medicine, National Yang-Ming University, Taipei, Taiwan

**Keywords:** complexity, sample entropy, multiscale entropy, bias, resting-state fMRI

## Abstract

Complexity analysis of resting-state blood oxygen level-dependent (BOLD) signals using entropy methods has attracted considerable attention. However, investigation on the bias of entropy estimates in resting-state functional magnetic resonance imaging (fMRI) signals and a general strategy for selecting entropy parameters is lacking. In this paper, we present a minimizing error approach to reduce the bias of sample entropy (SampEn) and multiscale entropy (MSE) in resting-state fMRI data. The strategy explored a range of parameters that minimized the relative error of SampEn of BOLD signals in cerebrospinal fluids where minimal physiologic information was present, and applied these parameters to calculate SampEn of BOLD signals in gray matter regions. We examined the effect of various parameters on the results of SampEn and MSE analyses of a large normal aging adult cohort (354 healthy subjects aged 21–89 years). The results showed that a tradeoff between pattern length *m* and tolerance factor *r* was necessary to maintain the accuracy of SampEn estimates. Furthermore, an increased relative error of SampEn was associated with an increased coefficient of variation in voxel-wise statistics. Overall, the parameters *m* = 1 and *r* = 0.20–0.45 provided reliable MSE estimates in short resting-state fMRI signals. For a single-scale SampEn analysis, a wide range of parameters was available with data lengths of at least 97 time points. This study provides a minimization error strategy for future studies on the non-linear analysis of resting-state fMRI signals to account for the bias of entropy estimates.

## Introduction

Since the inception of the resting-state blood oxygen level-dependent (BOLD) technique from functional magnetic resonance imaging (fMRI) (Biswal et al., 1995), an essential question emerged: What are the characteristics of the temporal dynamics of these seemingly noisy and spontaneous BOLD oscillations (Fox and Raichle, 2007)? The first piece of evidence is from the exhibition of 1/*f* frequency distribution of BOLD signals (Zarahn et al., 1997; Fox et al., 2007), which is an ubiquitous feature of the complex system (Schlesinger, 1987; Zang, 1991; Goldberger, 1996; Goldberger et al., 2002a), and has been observed in other neurophysiologic signals (Linkenkaer-Hansen et al., 2001; Stam and de Bruin, 2004). In complex systems, the 1/*f* noise is likely to arise from underlying oscillatory components operating at multiple time scales and is distinct with uncorrelated randomness (Zang, 1991; Hausdorff and Peng, 1996; Goldberger et al., 2002a); thus, using the entropy measure to assess the complexity of seemingly noisy physiologic data may provide hints for understanding the dynamics of a physiologic system (Pincus, 1991; Richman and Moorman, 2000), and for delineating the dynamical changes in physiologic systems of healthy and pathologic states (Costa et al., 2002; Goldberger et al., 2002b; Lipsitz, 2002; Peng et al., 2009).

**FIGURE 1:**
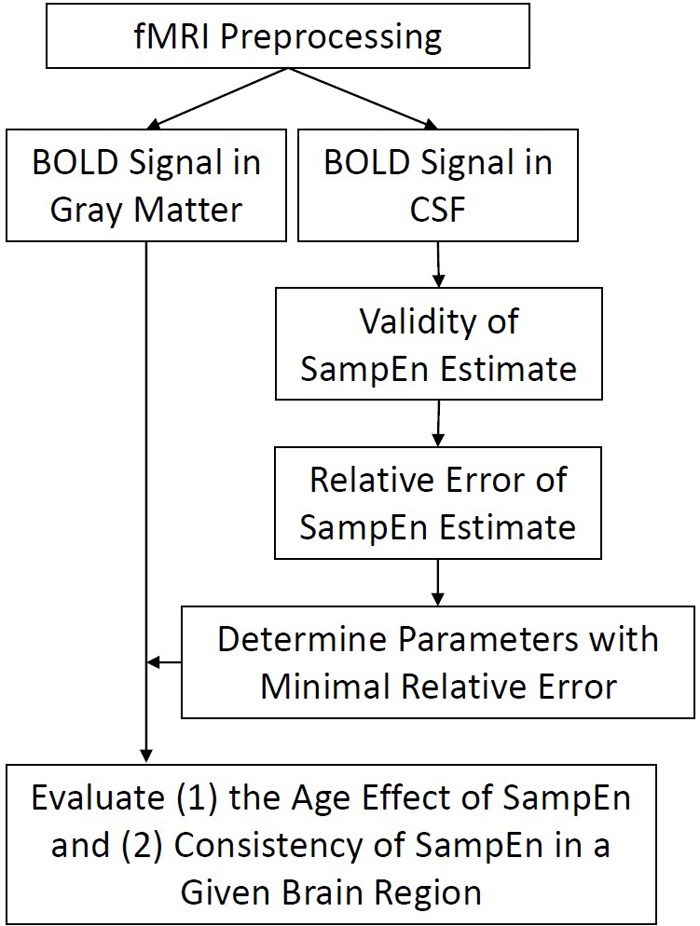
Flow chart of analyses. Functional imaging data were preprocessed and BOLD signal in every gray matter and CSF voxels was extracted for subsequent SampEn/MSE analysis. The evaluation of SampEn of BOLD signal is twofold. First, we evaluated the validity of SampEn calculation using a variety of combinations of parameters *m, r*, and Scale Factors. Second, we evaluated the relative error of BOLD signal using a minimization strategy. Finally, we applied the obtained parameters to study the effect of age in SampEn of gray matter voxels and evaluated the consistency of SampEn in a given brain region.

To develop a systemic approach for quantifying temporal dynamics of brain signal data in healthy and pathological states, we have proposed a loss of brain complexity hypothesis (**Figure 1**) to study mental and brain function in normal and pathological conditions (Yang and Tsai, 2013). The hypothesis is intuitively based on the observation that behavioral symptoms observed in patients often follow the pattern of order or randomness, and can be summarized as follows: (1) the complexity of a brain reflects its ability to adapt and function in an ever-changing environment, (2) brain operates across multiple scales of space (i.e., brain regions) and time (i.e., temporal changes), hence the complexity of brain oscillations is also multiscale and hierarchical, and (3) aging and a wide class of mental illness appear to reduce the adaptive capacity of the brain. Thus, loss of brain complexity may be a generic, defining feature of pathologic brain.

A variety of entropy measures has been applied to study the brain complexity by measuring temporal dynamics of fMRI signals. Some of these studies have used Shannon entropy (de Araujo et al., 2003; Leite and Mandeville, 2006; Goni et al., 2011; Tobia et al., 2012) and related families (Sturzbecher et al., 2009) to quantify the activated patterns of BOLD signals in various fMRI task experiments. For resting-state fMRI signals, a Wavelet entropy has been applied to study the resting-state complexity in schizophrenia (Bassett et al., 2012), and recently, we and others introduced multiscale sample entropy (MSE) to study the complexity of resting-state fMRI signals of normal aging (Yang et al., 2013a; Siero et al., 2014), the effect of genetic polymorphism on resting-state fMRI complexity (Yang et al., 2014), the characteristics of BOLD signals in various brain regions (McDonough and Nashiro, 2014), and psychosis (Yang et al., 2015; Hager et al., 2017). Other studies have also applied single-scale approximate entropy (ApEn) (Sokunbi et al., 2011; Liu et al., 2013) or sample entropy (SampEn) to study the resting-state fMRI signals of normal aging (Sokunbi, 2014), attention deficit hyperactivity disorder (Sokunbi et al., 2013), and schizophrenia (Sokunbi et al., 2014).

Among these entropy methods, SampEn and a related family, MSE, have attracted considerable attention because of their simplicity and the advantage of being less dependent on the time series length than ApEn. However, abundant results of entropy analyses of resting-state fMRI signals also come with the inconsistency of parameter selection for entropy calculation. The calculation of SampEn requires a tolerance factor *r* (typically a fraction of the standard deviation, SD, of a given signal) to determine the number of matches of data points using a pattern length *m*. Selections of *m* in SampEn are sometimes based on theoretical calculations for ApEn which suggest that 10*^m^* points should be sufficient, although 20*^m^*–30*^m^* points would be preferable for an accurate estimate (Pincus and Goldberger, 1994; Kirchner et al., 2012). However, there was no definite guideline to choose these parameters (Gow et al., 2015).

Generally, the selection of these parameters in fMRI studies have been based on maximizing the between-group difference in entropy estimates (Sokunbi et al., 2013, 2014; Yang et al., 2013a; Sokunbi, 2014), prior SampEn reports on other signals (Siero et al., 2014), or the conceptual notion that a sufficient pattern length was required to capture underlying dynamics (McDonough and Nashiro, 2014). Consequently, a variety of parameters have been reported, including *m* = 1, *r* = 0.35 (Yang et al., 2013a, 2014, 2015; Hager et al., 2017), *m* = 2, *r* = 0.3 (Siero et al., 2014; Sokunbi, 2014), *m* = 2, *r* = 0.32 (Sokunbi et al., 2014), *m* = 2, *r* = 0.46 (Sokunbi et al., 2013), or *m* = 2, *r* = 0.50 (McDonough and Nashiro, 2014).

The selection of SampEn parameters based on the approach of maximizing the between-group difference likely varies among studies and is not guaranteed to be more free from error or bias (McDonough and Nashiro, 2014). A general strategy for selecting SampEn parameters in resting-state fMRI signals is lacking. Although Lake et al. (2002) stated that one of the advantages of SampEn is its consistency, and that if one record showed lower SampEn than another with one set of *m* and *r* values, then it would also show lower SampEn with different parameters; however, the problems in the selection of SampEn parameters are not trivial because the bias of SampEn has not been explored in previous fMRI studies, and errors may influence neuroimaging studies because of the relatively short BOLD signals and large volume of brain voxels to be analyzed.

In the study of physiologic time series such as heart rate, we will observe a variance of entropy estimates that results from different physiologic conditions, age, sex, or the error of entropy estimate itself. Likewise, the temporal dynamics of BOLD signal across brain voxels is associated with local post-synaptic potentials in gray matter and action potentials in white matter (Gawryluk et al., 2014). However, such neuronal-related variance of entropy measures from BOLD signal in gray matter may be also contaminated by non-neuronal hemodynamic responses or the error of entropy calculation. Therefore, minimizing the error of entropy estimate could potentially maximize the reliability and consistency of quantification of neuronal-related entropy in BOLD signal.

In accordance with Lake et al. (2002) to minimize the bias of entropy calculation in heart rate, this study developed a generic strategy to minimize the relative error of SampEn calculation for resting-state fMRI signals. A range of parameters was examined to minimize the relative error of SampEn in cerebrospinal fluids (CSFs) that had minimal physiologic information, and then the appropriate SampEn parameters with low relative error were determined for use in gray matter regions. We investigated the effect of various parameters on the results of SampEn and MSE in a large normal aging cohort of resting-state fMRI datasets.

## Materials and Methods

### Participants

This study cohort comprised 354 healthy Han Chinese adult participants recruited from communities in Northern Taiwan (age range: 21–89 years; male/female: 185/169) (**Table 1**). The participants were selected from a larger cohort (502 subjects at the time of this study) based on a continuing effort of the Healthy Aging Project (Yang et al., 2013a, 2014) conducted in accordance with the Declaration of Helsinki. Approval was received from the institutional review board at Taipei Veterans General Hospital. Because we previously demonstrated that older subjects with Apolipoprotein-E (APOE) 𝜀4 genotype had reduced BOLD complexity compared with APOE 𝜀4 non-carriers (Yang et al., 2014), we did not include any APOE 𝜀4 carriers in this study.

**Table 1:** Normal aging cohort characteristics.

Age group (year)	No. of subjects	Females (%)	Total gray matter volume (cm^3^)
20–29	65	32 (49.2)	651 ± 55
30–39	46	23 (50.0)	624 ± 60
40–49	47	27 (57.4)	575 ± 47
50–59	61	35 (57.4)	574 ± 59
60–69	66	40 (60.6)	524 ± 48
70–79	28	9 (32.1)	475 ± 52
80–89	41	3 (7.3)	447 ± 45

Each participant was evaluated by a trained research assistant using a mini-international neuropsychiatric interview to exclude those with Axis I psychiatric disorders (Sheehan et al., 1998). Older participants (age >59 years) were further assessed using the Clinical Dementia Rating (CDR) scale (Hughes et al., 1982) to exclude those with dementia (CDR > 0). The overall exclusion criteria for all participants consisted of the following: (a) the presence of dementia; (b) the presence of Axis I psychiatric disorders, such as schizophrenia, bipolar disorders, or unipolar depression; and (c) a history of neurological conditions, such as head injury, stroke, or Parkinson’s disease.

### Image Acquisition and Processing

Functional magnetic resonance imaging was performed at National Yang-Ming University by using a 3.0T Siemens MRI scanner (Siemens Magnetom Tim Trio, Erlangen, Germany) equipped with a 12-channel head coil. The scanning protocol was consistent with our prior reports (Yang et al., 2013a, 2014, 2015). For resting-state image scanning, T2^∗^-weighted images with BOLD contrast were measured using a gradient echo-planar imaging (EPI) sequence (repetition time TR = 2,500 ms, echo time TE = 27 ms, FOV = 200 mm, flip angle = 77°, matrix size = 64 × 64, voxel size = 3.44 mm × 3.44 mm × 3.40 mm). For each run, 200 EPI volume images were acquired along the AC–PC plane. Structural T1 images were acquired with the 3D magnetization-prepared rapid gradient echo sequence (3D-MPRAGE; TR = 2,530 ms, TE = 3.5 ms, TI = 1,100 ms, FOV = 256 mm, flip angle = 7°). T1 images were segmented to estimate the total gray matter volume for each subject.

Resting-state fMRI data were preprocessed and analyzed using SPM8 (Wellcome Department of Imaging Neuroscience, London, United Kingdom) implemented in MATLAB ((MathWorks, Natick, MA, United States). The fMRI images were slice-time corrected, realigned, and normalized into the standard stereotaxic space of the Montreal Neurological Institute (MNI) EPI template, and resampled to a 3-mm cubic voxel. Covariates of the fMRI time series were regressed out, including the time courses of six head motion, white matter, and CSF. To avoid introducing distortions in the time series data, no global signal regression was performed (Murphy et al., 2009; Anderson et al., 2011). All participants included in this study exhibited a maximum displacement of less than 1.5 mm at each axis and an angular motion of less than 1.5° for each axis. The first five data points (12.5 s) in any fMRI time series were discarded because of the instability of the initial fMRI scanning, leaving 195 data points in the final data. Temporal low-pass filtering (0.01–0.08 Hz) was performed to reduce the influence of high-frequency noise from physiologic confounders.

### Sample Entropy and Multiscale Entropy Analysis

SampEn (Richman and Moorman, 2000) was developed to reduce the bias of a related family, ApEn (Pincus, 1991), and has a closer agreement with theoretical estimations than ApEn. Briefly, SampEn is defined by the negative natural logarithm of the conditional probability that a data set of length *N*, having repeated itself within a tolerance of *r* (similarity factor) for *m* points (pattern length), will also repeat itself for *m* + 1 points without allowing self-matches (Richman and Moorman, 2000). In practice, the number of matches of pattern length *m* within a tolerance of *r* was defined as *B*, and *A* was defined as the subset of *B* that also matched pattern length *m* + 1. Thus, SampEn was estimated by the negative natural logarithm of the ratio CP = *A*/*B* that SampEn = -log CP.

SampEn is a measure of regularity based on a single and shortest time scale (Richman and Moorman, 2000; Lake et al., 2002). Such a single-scale entropy measure produces higher values of entropy to uncorrelated noise, which is presumed to convey less information than 1/*f* noise (Goldberger et al., 2002a,b). Consequently, the MSE analysis (Costa et al., 2002) was introduced to estimate the entropy on multiple time scales based on the notion that complex dynamics typically arise from multiple time scales and that a generic approach to measure global complexity must account for the multiple time scales in a given physical system (Zang, 1991; Fogedby, 1992). The MSE calculation can be summarized in three steps: (1) construct a coarse-grained time series according to a range of scale factors, (2) quantify the SampEn of each coarse-grained time series, and (3) examine the MSE profile by using a range of scales. The length of each coarse-grained time series is equal to the length of the original time series divided by the scale factor. For Scale 1, the time series was simply the original time series.

### A General Strategy for Selecting Parameters for the SampEn/MSE Analysis of fMRI Signals

As mentioned, three parameters were involved in the SampEn/MSE analysis, including the pattern length *m*, tolerance factor *r*, and the time scale factor. In principle, a sufficient pattern length *m* and a small *r* value is ideal for capturing underlying dynamics when the irregularity of a given signal is increased (Pincus, 1991). However, in practice, the confidence of the SampEn estimation was dependent on the number of pattern matches for lengths *m* and *m* + 1 (i.e., *A* and *B*). The stringent criteria for a large *m* and small *r* resulted in fewer pattern matches, and thus increased the statistical variation in calculating CP (i.e., *A*/*B*) and SampEn. By contrast, a relaxed criterion for a small *m* and large *r* resulted in more pattern matches in both *A* and *B*, thus causing the SampEn value to be close to 0 and reducing the ability of SampEn to discriminate dynamical processes (Lake et al., 2002).

Lake et al. (2002) proposed a general strategy to appropriately select *m* and *r* by (a) selecting *m* by using the autoregressive (AR) model order for a given signal and (b) minimizing the relative error of the SampEn calculation. In their study, the relative error of SampEn was estimated theoretically and applied to 200 randomly selected cardiac R-R interval time series (4096 data points). However, such a theoretical estimation is computationally exhausting and is unlikely to be practical for use with large amounts of resting-fMRI BOLD signals. Therefore, we adopted Lake et al.’s (2002) principle but used a straightforward strategy.

Empirically, the SampEn of BOLD signals can be computed directly in all brain voxels and the variance of SampEn can subsequently be estimated. However, the SampEn variance in gray matter contains not only error but also critical information related to neuronal signal dynamics. A direct minimization of the SampEn variance in the gray matter region will likely reduce the ability of SampEn to discriminate brain processes. By contrast, BOLD signals in CSFs have been considered as a nuisance and are routinely regressed out for contaminating gray matter BOLD signals (Biswal et al., 1995, 1997). Furthermore, recent reports showed that CSF signals exhibited the characteristics of uncorrelated noise (Wu et al., 2012; McDonough and Nashiro, 2014), thus opening the possibility of using CSF BOLD signals as the random control to minimize the bias of SampEn and to determine appropriate SampEn parameters.

Therefore, a general strategy was developed to explore a range of parameters that minimized the relative error of SampEn of BOLD signals in CSFs; the obtained parameters were then applied to study the SampEn of BOLD signals in gray matter. This minimization strategy considers the distinct BOLD signal properties between CSFs and gray matter and is presumed to be consistent across studies; thus, problems in prior approaches that maximize the between-group difference of entropy estimates in gray matter regions are avoided. In addition, a selection of pattern length *m* may be beneficial by studying the AR model order of the underlying structure of BOLD signals in gray matter, which is the primary brain region with functional relevance. However, we decided that this approach was less critical because the ability of SampEn to capture underlying dynamics is dependent not only on the pattern length *m*, but also on the tolerance factor *r*. Therefore, the selection of *m* and *r* in this study should be primarily based on minimizing the relative error of SampEn.

Adopting the methods proposed by Lake et al. (2002), we defined the relative error of SampEn as the 95% confidence interval (CI) of the SampEn estimate relative to the SampEn value. A relative error of 0.05 corresponds to a 95% CI that is 10% of the SampEn estimate (Lake et al., 2002). This relative error can be empirically estimated by calculating the mean and SD of SampEn of BOLD signals in all CSF voxels in a subject (i.e., 1.96 ×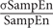/2). This relative error metric is approximately the same as the coefficient of variation (CV), which is a measure of the dispersion of SampEn distribution. Because of short BOLD data (195 data points compared to 4096 RR intervals in Lake et al., 2002), we aimed for a relative error no higher than 0.1, which was approximately 10% of the CV value in SampEn estimates.

### Statistical Analysis

A flow chart of analysis involved in this paper was shown in **Figure 1**. Briefly, functional imaging data were preprocessed and BOLD signal in every gray matter and CSF voxels was extracted for subsequent SampEn/MSE analysis. The evaluation of SampEn of BOLD signal is twofold. First, we evaluated the validity of SampEn calculation using a variety of combinations of parameters *m, r*, and Scale Factors. The validity of SampEn indicated if a SampEn value can be derived from short BOLD signal using a given set of parameters. Second, we evaluated the relative error of BOLD signal using the aforementioned minimization strategy. Finally, we applied the obtained parameters to study the effect of age in SampEn/MSE of gray matter voxels and evaluated the consistency of SampEn in a given brain region.

The relative error was obtained from the CSF region of each subject, and a median value of the relative error of all subjects was reported for a given *m* and *r*. A CSF mask provided by a REST toolbox that contained 121 CSF voxels (3 mm × 3 mm × 3 mm) (Song et al., 2011) was used in this study. To maintain the consistency of fMRI signal characteristics across all brain voxels, we used postprocessed BOLD image data and normalized each BOLD time series for a zero mean and unit SD before conducting the SampEn/MSE analysis. We assess the relative error of SampEn for a wide range of combinations of *m* and *r*, and to examine the effect of BOLD data length (coarse-grained BOLD time series by various scale factors) on the relative error of SampEn. We also examined the AR model order for all gray matter voxels in the entire study cohort.

After determining a range of appropriate parameters for SampEn/MSE analyses, we applied these parameters to the SampEn calculation of BOLD signals in all gray matter voxels in each subject. A general linear model (GLM) controlling the effect of sex and total gray matter volume on SampEn was used to examine the primary effect of age on BOLD SampEn data. We used the GLM separately for the BOLD SampEn data of each scale factor, as well as for the overall average SampEn across all scale factors. We also compared the results of the GLM using various sets of SampEn parameters and evaluated the CV of t-statistics across gray matter voxels in a given brain region as a proxy of the consistency of SampEn calculation. Significant brain clusters with peak coordinates in the MNI space were reported if the *p*-value corrected for the family-wise error rate was less than 0.05 at the cluster level.

## Results

### Characteristics of the SampEn/MSE Analysis

**FIGURE 2:**
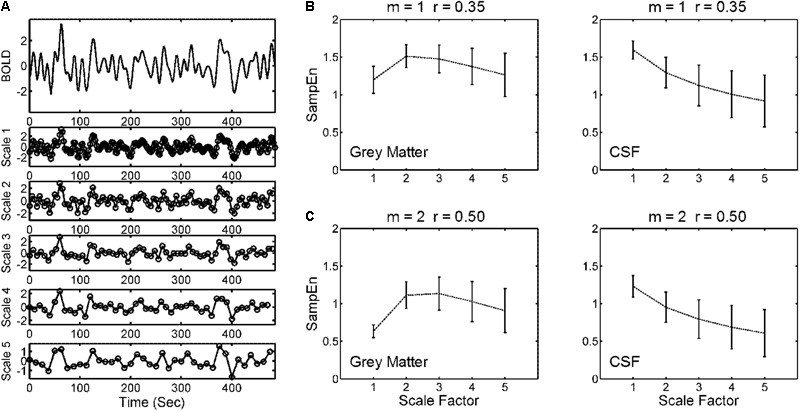
Illustration of a multiscale sample entropy (SampEn; MSE) analysis. **(A)** Coarse-graining of a BOLD time series from a gray matter voxel of an individual was performed by averaging the data points within non-overlapping windows of increasing lengths of Scale Factors 1–5. Sample entropy for each scale factor was estimated from the coarse-grained time series. **(B)** Mean and 95% CI of MSE profiles across Scale Factors 1–5 from (left) all gray matter voxels and (right) all CSF voxels of the entire study cohort (354 subjects). The parameters for SampEn calculation were pattern length *m* = 1 and tolerance factor *r* = 0.35. **(C)** The same analysis as **(B)** but with distinct SampEn parameters (*m* = 2, *r* = 0.50). The strategy was to explore a range of parameters that minimized the relative error of SampEn of BOLD signals in CSFs where minimal physiologic information was present, and to apply these parameters to calculate the SampEn of BOLD signals in gray matter regions.

**Figure 2A** illustrates the coarse-graining of the BOLD time series in the MSE analysis. The coarse-graining averaged the data points within non-overlapping windows of increasing lengths of Scale Factors 1–5. SampEn for each scale factor was estimated from the coarse-grained time series. **Figures 2B,C** show the profile of SampEn from Scale Factors 1 to 5 averaged across all gray matter and CSF voxels in the entire study cohort, from using SampEn parameters reported in prior studies (*m* = 1, *r* = 0.35 and *m* = 2, *r* = 0.50). The mean SampEn across various scales revealed a consistent pattern with distinct parameters of *m* and *r*, but the 95% CI of SampEn increased with increasing scale factors.

### The Valid Parameters for the SampEn/MSE Analysis in BOLD Signal

First, we examined how data length and selection of parameters *m* and *r* could result in invalid SampEn estimates due to the absence of pattern matches in BOLD signals. The absence of pattern matches could be due to short data length (i.e., lack of sufficient data sample for finding a match), large pattern length *m* (i.e., lack of the recurrence of complex pattern), or small *r* (i.e., unable to find a match within a narrow similarity criterion). We performed the experiment by calculating SampEn in CSF BOLD signals using a variety of combinations of *m, r* and Scale Factors.

**FIGURE 3:**
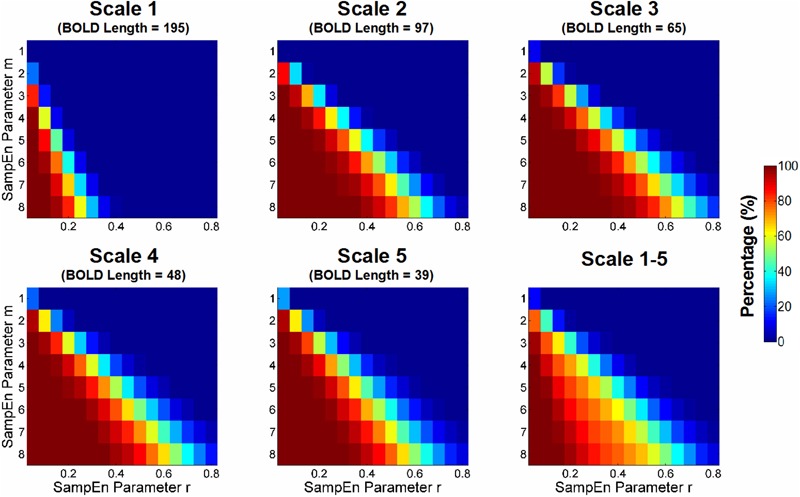
Percentage of estimation failure for sample entropy (SampEn) because of the absence of pattern matches in CSF BOLD signals. The percentage was calculated based on the CSF voxels with invalid SampEn relative to all CSF voxels in the entire study cohort. The percentage was compared with those of various combinations of *m* and *r* across all scale factors with different BOLD signal lengths. The more stringent combinations of *m* and *r* (i.e., higher *m* and lower *r*) and shorter BOLD signals resulted in a higher percentage of invalid SampEn estimations. Ideally, the choice of *m* and *r* should have valid SampEn estimations in all voxels.

**Figure 3** shows the percentage of SampEn estimation failures that were caused by the absence of pattern matches in CSF BOLD signals. The percentage was calculated based on the CSF voxels with invalid SampEn estimates relative to all CSF voxels in the entire study cohort. The results showed that more stringent combinations of *m* and *r* (i.e., higher *m* and lower *r*) and shorter BOLD signals resulted in a higher percentage of invalid SampEn estimates. Ideally, a combination of *m* and *r* should be selected only when the BOLD signals of all voxels have valid SampEn estimates. Therefore, a range of combinations of *m* and *r*, including *m* = 1 and *r* ≥ 0.20, *m* = 2 and *r* ≥ 0.35, *m* = 3 and *r* ≥ 0.60, and *m* = 4 and *r* ≥ 0.75, was free for invalid SampEn calculation.

### Estimation of the Relative Error of the SampEn/MSE Analysis

As aforementioned, the minimization of the relative error of SampEn was performed in CSF BOLD signals that contained minimal physiologic information and exhibited the characteristics of uncorrelated noise. The relative error of SampEn was empirically estimated by calculating the mean and SD of SampEn of BOLD signals in all CSF voxels in every subject. The relative error measured the dispersion of SampEn distribution in CSF regions, thereby provide a metric to evaluate the bias of SampEn estimates because the variance of entropy in CSF BOLD signal is presumably consistent across CSF voxels.

**FIGURE 4:**
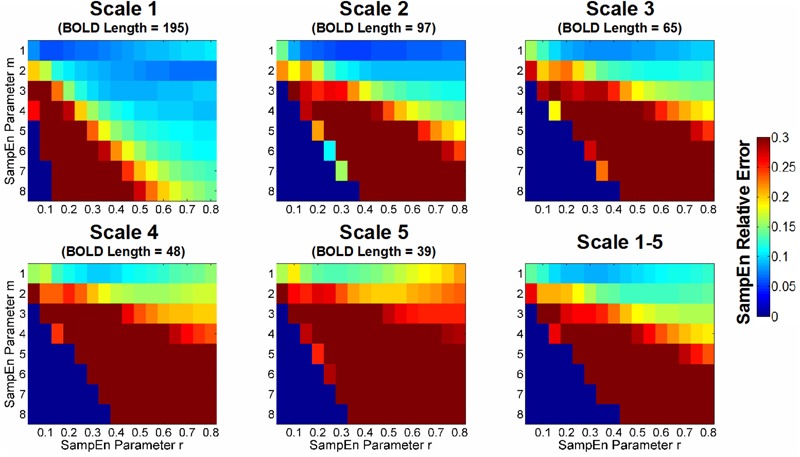
Color map of the relative error of sample entropy (SampEn) of CSF BOLD data for appropriate selection of *m* and *r*. The SampEn relative error was defined in accordance with Lake et al. (2002) based on the 95% CI relative to the average SampEn in all CSF voxels; thus, the metric indicated the consistency of the SampEn calculation. A lower SampEn relative error indicated a higher consistency of SampEn. For example, a SampEn relative error value of 0.05 corresponds to a 95% CI, which is 10% of the average SampEn estimate. In this study, the goal was to minimize the relative error of SampEn in CSF BOLD signals because they exhibit characteristics of uncorrelated randomness and contain minimal physiologic information. The median value of the SampEn relative error for the entire study cohort is shown in color with various combinations of *m, r*, and scale factors.

**Figure 4** shows the color map of the relative error of SampEn calculation in CSF BOLD signals. The lower SampEn relative error indicates a higher consistency (i.e., lower variation) of SampEn among the CSF voxels. We set the criteria for the selection of *m* and *r* to have a relative error lower than 0.1. For Scale 1 (BOLD length = 195 time points), the acceptable range of *m* and *r* was *m* = 1, 0.05 ≤*r* ≤ 0.70, *m* = 2, 0.25 ≤*r* ≤ 0.80, *m* = 3, 0.35 ≤*r* ≤ 0.80, and *m* = 4, 0.55 ≤*r* ≤ 0.80. For Scale 2 (BOLD length = 97 time points), the acceptable range of *m* and *r* was *m* = 1, 0.10 ≤*r* ≤ 0.80, and *m* = 2, 0.40 ≤*r* ≤ 0.80. For Scale 3 (BOLD length = 65 time points), the acceptable range of *m* and *r* was *m* = 1, 0.15 ≤*r* ≤ 0.80. For Scale 4 (BOLD length = 48 time points), the acceptable range of *m* and *r* was *m* = 1, 0.30 ≤*r* ≤ 0.35. For Scale 5 (BOLD length = 39 time points), there was no error rate of SampEn below 0.1 for any *m* and *r*.

For the average relative error of Scales 1–5, the only range with an acceptable SampEn error was *m* = 1, 0.20 ≤*r* ≤ 0.45. The optimal *m* and *r* in this range was *m* = 1, *r* = 0.30 (relative error = 0.087). When *m* ≥ 2, the minimum error rate was beyond 0.1 for *m* = 2, *r* = 0.55 (relative error = 0.128) and *m* = 3, *r* = 0.70 (relative error = 0.162). When *m* ≥ 4, the minimum error rate was beyond 0.2.

For comparison, we chose three sets of parameters with increasing levels of relative error: *m* = 1, *r* = 0.35 (relative error = 0.089), *m* = 2, *r* = 0.50 (relative error = 0.129), and *m* = 3, *r* = 0.70 (relative error = 0.162). The first two chosen sets of parameters were consistent with prior reports (Sokunbi et al., 2013; Yang et al., 2013a; McDonough and Nashiro, 2014) and were close to the minimum of relative error for a given *m*. Although the AR model order suggested a choice of *m* ≥ 3, the error rate for *m* ≥ 3 was beyond the acceptable error rate.

### Effect of Entropy Parameters on the SampEn/MSE Analysis of BOLD Signals in Normal Aging Data

**FIGURE 5:**
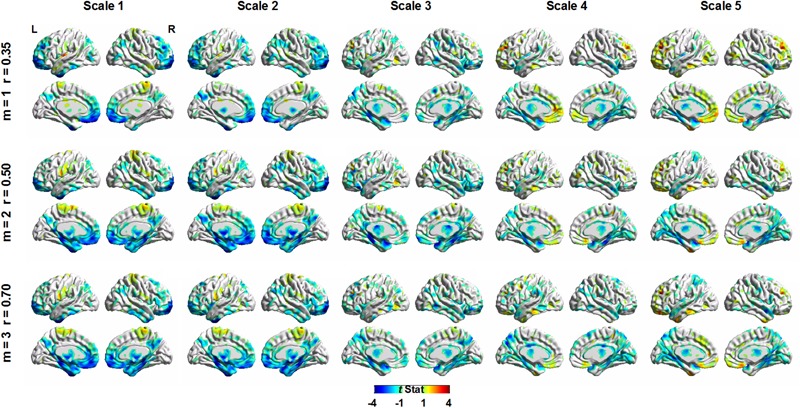
Voxel-wise correlation between age and multiscale entropy using a general linear model (GLM) to control the effect of sex and total gray matter volume. The GLM was used separately for sample entropy parameters of *m* = 1, *r* = 0.35; *m* = 2, *r* = 0.50; and *m* = 3, *r* = 0.70.

**Figure 5** shows the voxel-wise correlation between age and MSE using the GLM to control the effect of sex and total gray matter volume on MSE values. The GLM was used separately for SampEn parameters of *m* = 1, *r* = 0.35; *m* = 2, *r* = 0.50; and *m* = 3, *r* = 0.70. For all three parameters, visual inspection of MSE brain topography suggested a similar pattern of brain regions with negative correlations between age and MSE. The results from parameters *m* = 2, *r* = 0.50, and *m* = 3, *r* = 0.70 showed larger brain clusters with negative correlations between age and MSE than parameter *m* = 1, *r* = 0.35.

**FIGURE 6:**
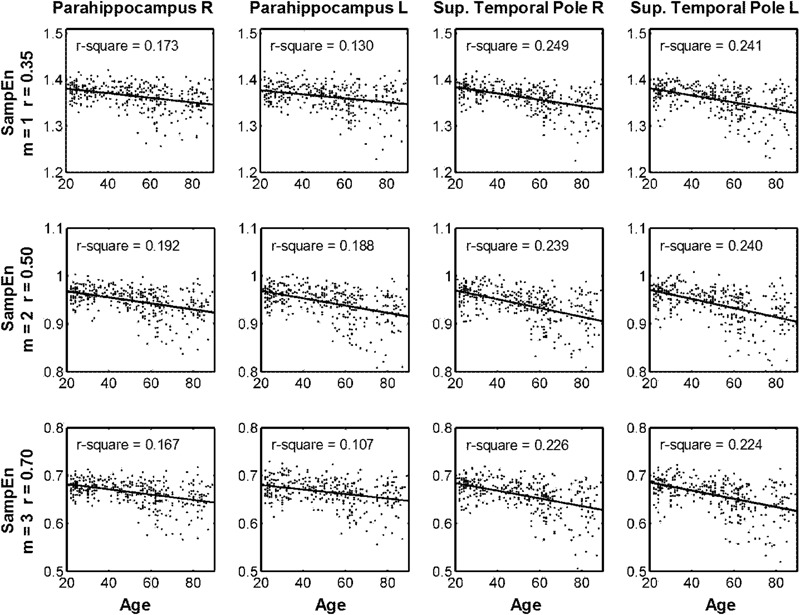
Scattered plots of correlation between age and multiscale entropy (MSE) based on the average sample entropy (SampEn) with various parameters calculated in the four brain regions identified in **Table 2**.

**Table 2** summarizes the statistical results by using the average MSE value of Scales 1–5. The results from the three parameters showed the same brain regions with significant negative correlations between age and MSE values, including the right and left parahippocampus and right and left superior temporal pole. Although the largest brain clusters and the strongest peak *t* value were found in the results of using *m* = 2, *r* = 0.50, there was no significant difference in the mean *t* value when comparing the *t* statistics of the same brain regions examined by using various parameters. Furthermore, the CV of *t* statistics within a given brain region was lower in the results of using *m* = 1, *r* = 0.35 than those in the results of using the other parameters, suggesting a higher consistency of brain voxels identified by SampEn using *m* = 1, *r* = 0.35. We averaged the MSE value within identified brain clusters; **Figure 6** shows the scattered plots with a consistent pattern of correlation between age and the average MSE using various parameters.

**Table 2:** Regions showing significant correlation of age with multiscale entropy in the normal aging cohort.

Brain region^a^	BA	MNI coordinates (mm)			Volume (mm^3^)^b^	Peak *t*	Mean *t*	CV
		*x*	*y*	*z*				
***m* = 1, *r* = 0.35**					
Parahippocampus R		12	-30	0	1,404	-3.65	-3.27	0.039
Parahippocampus L		-21	-30	-12	1,404	-3.81	-3.35	0.052
Superior temporal pole R	38	42	-3	-15	2,781	-3.99	-3.46	0.057
Superior temporal pole L	38	-42	18	-21	2,943	-3.99	-3.40	0.054
***m* = 2, *r* = 0.50**					
Parahippocampus R		21	-30	-21	4,725	-3.84	-3.39	0.057
Parahippocampus L		-21	-30	-15	5,994	-4.62	-3.63	0.098
Superior temporal pole R	38	42	12	-18	6,588	-4.58	-3.62	0.102
Superior temporal pole L	38	-36	9	-21	5,157	-4.40	-3.52	0.084
***m* = 3, *r* = 0.70**					
Parahippocampus R		18	-36	-6	2,268	-3.71	-3.33	0.045
Parahippocampus L		-9	-27	6	4,239	-4.00	-3.40	0.068
Superior temporal pole R	38	42	0	-15	4,374	-4.60	-3.58	0.100
Superior temporal pole L	38	-36	9	-21	2,565	-4.01	-3.37	0.091

*^*a*^L, left; R, right; BA, Brodmann area; CV, coefficient of variation. ^*b*^Volume was computed from cluster size (3 mm × 3 mm × 3 mm voxel). All results had *p*-value less than 0.05 corrected for multiple comparisons using familywise error.*

## Discussion

We systemically evaluated the relative error of SampEn in a wide range of pattern length *m*, tolerance factor *r*, and various time scales. The strategy was to minimize the relative error of SampEn in CSFs where minimal physiologic information was present, and determine appropriate SampEn parameters to be used in gray matter regions. Our estimations provided an array of parameters *m* and *r* in various scales of BOLD signals with relative errors below 0.1. In general, a tradeoff between *m* and *r* was necessary to maintain the accuracy of SampEn calculation. In other words, an increased *m* value had to accompany an increased *r* value to maintain an acceptable error level in short fMRI time series.

For comparison, we chose *m* = 1, *r* = 0.35; *m* = 2, *r* = 0.50; and *m* = 3, *r* = 0.70 with increasing levels of error to evaluate the effect of SampEn parameters on the resting-state fMRI entropy analysis of a normal aging cohort. Qualitatively, the results from these three parameters consistently showed that the same brain regions had a significant negative correlation between age and SampEn at various time scales. These brain regions included the parahippocampus and superior temporal pole at both hemispheres. Quantitatively, *m* = 1, *r* = 0.35 resulted in smaller but more consistent brain clusters in terms of the CV value of *t* statistics. Larger brain clusters but also a reduced consistency of *t* statistics were shown in the results of using *m* = 2, *r* = 0.50, and *m* = 3, *r* = 0.70. These results suggested that an increased error of SampEn had a negative impact on the quantitative results of voxel-wise statistics, despite the qualitative results being the same in a large cohort. We expect that the adverse impact of such error on qualitative results will become apparent in a smaller dataset.

Overall, the parameters *m* = 1, *r* = 0.20–0.45 provided reliable MSE estimates for most scale factors, and the minimum error was found at *m* = 1, *r* = 0.30 for MSE analysis. For a single-scale SampEn analysis, a wide range of parameters is available with data lengths of at least 97 time points. We suggest that future studies on the complexity analysis of resting-state fMRI signals account for the relative error of SampEn. Our minimization strategy can also be generalized to other time domains and non-linear measures for fMRI data.

### Strategies for Selecting SampEn and MSE Parameters

Few strategies exist for assessing the parameters of ApEn, SampEn, and even MSE analyses, and most of these strategies were developed for ApEn. In general, statistical estimates of conditional probabilities become less reliable as *m* increases, and the loss of system dynamics information also increases as *r* increases (Pincus and Goldberger, 1994). The early study of ApEn in cardiac R-R intervals established a guideline for selecting parameters of data length ≥100, *m* ≤ 3, and *r* = 0.1–0.25 of SD of input data (Pincus and Goldberger, 1994). Many studies have arbitrarily adopted the parameter *m* = 2, *r* = 0.1–0.2 to ApEn, SampEn, and MSE analyses (Costa et al., 2002; Alcaraz et al., 2010; Yentes et al., 2013).

These commonly used parameters were typically applied to signals with slower dynamics such as heart rate; hence, some studies have suggested that these parameters are inappropriate for signals with faster dynamics, and proposed to use *r* values that maximize the ApEn value (Chen et al., 2005; Chon et al., 2009). However, this maximum entropy approach was shown to be invalid for SampEn estimates (Castiglioni et al., 2013).

Lake et al. (2002) proposed a minimizing error approach for SampEn and found that *m* = 3, *r* = 0.2 was optimal for the cardiac R-R intervals at 4,096 time points. Lake et al.’s (2002) study also demonstrated a wide range of parameters with SampEn estimates that were within the acceptable error range, such as *r* = 0.1–0.8 for *m* = 1, and *r* = 0.2–0.5 for *m* = 2. These findings emphasized the advantage of SampEn for maintaining low error and consistency in a wide range of parameters.

Another approach is to maximize the differential ability of the entropy estimates for a certain dataset, such as finding optimal SampEn parameters to predict the termination and outcome of atrial fibrillation (Alcaraz et al., 2010). We and others also used similar approaches to maximize the ability of SampEn to differentiate the BOLD MSE between the older subjects with low and high cognitive scores (Yang et al., 2013a), and to differentiate healthy and ill subjects in various populations (Sokunbi et al., 2013, 2014; Yang et al., 2013b).

An obvious shortcoming of this approach is that the choices of parameters will be dependent on study populations. Furthermore, maximizing the between-group difference does not guarantee that those parameters are more free from error or bias (McDonough and Nashiro, 2014). The minimizing error approach we adopted from Lake et al. (2002) can eliminate the problems of the maximizing between-group difference approach.

### Is a Larger Pattern Length *m* Superior to a Smaller *m* to Capturing Signal Dynamics?

Our results showed that there was no substantial difference in brain regions detected by SampEn or MSE analyses using *m* = 1, 2, or 3. This observation contradicted the results of the AR model and the long-standing idea that a choice of a larger *m* is superior to smaller *m* because it provides a more detailed reconstruction of system dynamics (Pincus and Goldberger, 1994; Groome et al., 1999; Lake et al., 2002). This notion formed when the development of entropy measures was influenced by the theories of phase space and embedding dimensions (Takens, 1981), and the empirical evidence for the notion was based on a relatively long time series (such as 4,096 data points in Lake et al., 2002), which allowed sufficient statistics of complex dynamics. Our findings suggested that this notion was compromised in short time series because a small *m* (i.e., *m* = 1) was as sufficient to capture the dynamics of short BOLD signals as *m* = 2 or 3. These results are similar to a prior report that a small *m* = 1 was sufficient to detect atrial fibrillation in short heartbeat time series (Lake and Moorman, 2011). Furthermore, we found that the effect of chosen parameters primarily reflected the SampEn error and quantitative results of brain clusters, suggesting that the effect of error in entropy estimates may outweigh the importance of selecting *m* and *r* for the reconstruction of underlying dynamics in short time series.

The choice of *m* = 1 and *r* = 0.35 in our data did not prevent the use of different parameters from other resting data with higher scanning volumes. For long resting-state fMRI time series (e.g., 1,200 time points) such as those from the Human Connectome Project (Van Essen et al., 2013), we suggested that the parameter *m* = 2, *r* = 0.50 may be too relaxed (McDonough and Nashiro, 2014), and we anticipate that *m* = 2 or 3 (according to the AR model) and *r* < 0.5 may help to uncover subtle dynamics in long-term resting data that are not otherwise apparent.

### Time Series Length Constraints on Selecting Pattern Length *m* in SampEn

A critical but often overlooked parameter in entropy estimates is the time series length constraints. The accuracy of entropy estimates is dependent on the data length to accrue sufficient statistics. Such data length constraints limit the selection of pattern length *m* because a large *m* will increase the chance of bias in entropy calculation. The theoretical work of time series length constraints on selecting *m* has been documented in ApEn that A time series with a length of at least 10^m^ to 20^m^ is necessary to obtain reliable ApEn estimates (Pincus and Goldberger, 1994). SampEn was developed to improve the consistency of entropy estimates in various data lengths (Richman and Moorman, 2000), and is therefore less vulnerable to time series length constraints than ApEn. However, Richman and Moorman (2000) also found that SampEn was unreliable for data lengths below 100 time points when using *m* = 2 (i.e., but the time series length effect with *m* = 1 was not tested). One recent study suggested that both ApEn and SampEn are extremely sensitive to parameter choices for short data sets ≤200 time points (Yentes et al., 2013). Because of the similarity between SampEn and ApEn, we adopted the same theoretical criteria of ApEn to estimate the time series length and pattern length *m* in the MSE analysis of short BOLD signals (Yang et al., 2013a).

While such a strategy may be questionable (Sokunbi, 2014), our results of relative error of SampEn in various scale factors may validate its reliability. The maximum pattern length *m* with a relative error below 0.1 for each scale factor was: *m* = 4 for Scale 1 (195 time points), *m* = 2 for Scale 2 (97 time points), *m* = 1 for Scales 3 and 4 (65 and 48 time points, respectively), and no pattern length *m* was able to maintain a relative error below 0.1 for Scale 5 (39 time points). The pattern of these results clearly suggested that SampEn was not subject to the theoretical constraints of ApEn (10^m^–20^m^) for data with at least 195 time points (i.e., *m* can be up to 4 in Scale Factor 1 with 195 time points). However, in a much shorter time series below 97 time points, the choices of pattern length *m* of SampEn may resemble that of ApEn and may be vulnerable to time series constraints.

Our results suggested that at a data length of approximately 97 time points, an *r* value larger than 0.4 was required for *m* = 2 to maintain a relative error below 0.1; this result was similar to the experimental data collected by Richman and Moorman (2000), using a data length 100 time points. Hence, although a resting-state fMRI study using single-scale SampEn may have a wide range of *m* and *r* available in short time series of at least 97 data points (**Figure 3**), the MSE analysis with a coarse-graining procedure has to consider these time series length constraints.

### Effect of Normal Aging on Resting-State Brain Complexity

The normal aging data presented in this study were used as an attempt to improve our previous study (Yang et al., 2013a), which did not consider the between-age-group difference in the MSE analysis of the effect of gray matter volume loss in older people. Although we briefly studied this issue when examining the effect of APOE 𝜀4 on MSE complexity (Yang et al., 2014), that study was based on younger and older groups and cannot be generalized to the adult lifespan. After regressing out the effect of age-related linear decline in total gray matter volume on MSE, the parahippocampus and superior temporal pole still showed a significant decline in MSE with increasing age, suggesting that a volume-independent functional change occurred in these two brain areas. Because both areas had neuropathologic changes caused by normal aging and Alzheimer’s disease (Arnold et al., 1994; Mitchell et al., 2002), whether a reduced MSE in these areas can be explained by the accumulation of neurofibrillary tangles warrants further.

### Limitations

This study was subject to certain limitations. First, the minimization strategy focused exclusively on the CSF region. Some studies have suggested that white matter also exerts some influences through noise (Liu et al., 2013; Siero et al., 2014). Second, neuronal activity is not always associated with changes in cerebral blood flows (Huo et al., 2014); thus, additional studies are required to examine the extent of neuronal activity that contributes to the complexity of BOLD fMRI data. Third, the removal of physiologic noise may be improved by a more advanced approach, such as component-based noise correction method (Behzadi et al., 2007). Future study is also warranted to explore the relationship between the bias of entropy calculation and mitigation of physiologic noise. Fourth, the relative error used in Lake et al. (2002) was consistent with the CV. We used a similar metric, but because our study showed that a CV with a threshold of 10% was approximately the same as a relative error of 0.1, using a CV to judge the bias of SampEn in future studies directly might be easier for interpretation. Finally, MSE is not merely a calculation of SampEn on multiple time scales (Peng et al., 2009). The substantial difference between MSE and single-scale SampEn in distinguishing complexity and irregularity was beyond the scope of this study and warrants a systemic investigation, using normal aging and disease population data.

## Conclusion

We developed a general strategy to study the bias of the SampEn/MSE analysis of resting-state fMRI data and comprehensively examined the effect of various parameters on the relative error of SampEn estimates. Our results addressed the problems in the maximizing between-group difference approach and revealed a range of appropriate parameters that can be used in future resting-state fMRI studies with various data constraints. Finally, we expect that the bias minimization strategy can be generalized to other method of quantifying temporal dynamics of BOLD signal and improve the consistency of these methods to study abnormal brain activity in various brain diseases.

## Ethics Statement

This study was carried out in accordance with the recommendations of the institutional review board at Taipei Veterans General Hospital, with written informed consent from all subjects. All subjects gave written informed consent in accordance with the Declaration of Helsinki. The protocol was approved by the institutional review board at Taipei Veterans General Hospital.

## Author Contributions

AY designed the experiment, analyzed the data, performed the statistical analyses, and wrote the paper. C-PL provided MRI resource and technical support of the experiment. S-JT and C-KP contributed the materials and critically commented the manuscript. All authors have approved the final article.

## Conflict of Interest Statement

The authors declare that the research was conducted in the absence of any commercial or financial relationships that could be construed as a potential conflict of interest.
